# Coronary Artery Disease Diagnosis; Ranking the Significant Features Using a Random Trees Model

**DOI:** 10.3390/ijerph17030731

**Published:** 2020-01-23

**Authors:** Javad Hassannataj Joloudari, Edris Hassannataj Joloudari, Hamid Saadatfar, Mohammad Ghasemigol, Seyyed Mohammad Razavi, Amir Mosavi, Narjes Nabipour, Shahaboddin Shamshirband, Laszlo Nadai

**Affiliations:** 1 Department of Computer Engineering, Faculty of Engineering, University of Birjand, Birjand 97175/615, Iran; Javad.hassannataj@birjand.ac.ir (J.H.J.); saadatfar@birjand.ac.ir (H.S.); ghasemigol@birjand.ac.ir (M.G.); 2 Department of Nursing, School of Nursing and Allied Medical Sciences, Maragheh Faculty of Medical Sciences, Maragheh, Iran; edris.hn2000@gmail.com; 3 Department of Electronics, Faculty of Electrical and Computer Engineering, University of Birjand, Birjand 9717434765, Iran; smrazavi@birjand.ac.ir; 4 Kalman Kando Faculty of Electrical Engineering, Obuda University, 1034 Budapest, Hungary; amir.mosavi@kvk.uni-obuda.hu (A.M.); nadai@uni-obuda.hu (L.N.); 5 Institute of Structural Mechanics, Bauhaus Universität-Weimar, D-99423 Weimar, Germany; 6 Department of Mathematics and Informatics, J. Selye University, 94501 Komarno, Slovakia; 7 Faculty of Health, Queensland University of Technology, 130 Victoria Park Road, Queensland 4059, Australia; 8 Department Institute of Research and Development, Duy Tan University, Da Nang 550000, Vietnam; 9 Department for Management of Science and Technology Development, Ton Duc Thang University, Ho Chi Minh City, Vietnam; 10 Faculty of Information Technology, Ton Duc Thang University, Ho Chi Minh City, Vietnam

**Keywords:** heart disease diagnosis, coronary artery disease, machine learning, health informatics, data science, big data, predictive model, ensemble model, random forest, industry 4.0

## Abstract

Heart disease is one of the most common diseases in middle-aged citizens. Among the vast number of heart diseases, coronary artery disease (CAD) is considered as a common cardiovascular disease with a high death rate. The most popular tool for diagnosing CAD is the use of medical imaging, e.g., angiography. However, angiography is known for being costly and also associated with a number of side effects. Hence, the purpose of this study is to increase the accuracy of coronary heart disease diagnosis through selecting significant predictive features in order of their ranking. In this study, we propose an integrated method using machine learning. The machine learning methods of random trees (RTs), decision tree of C5.0, support vector machine (SVM), and decision tree of Chi-squared automatic interaction detection (CHAID) are used in this study. The proposed method shows promising results and the study confirms that the RTs model outperforms other models.

## 1. Introduction

Today, the healthcare industry has accumulated big data [1]. Data science has been greatly empowering the advancement of novel technologies for bringing insight into big data for smart diagnose, disease prevention, and policy-making in healthcare industry [2,3]. Among the data science technologies, machine learning for big data has been reported as the most effective strategies with an ever-increasing popularity in a broad range of applications in preventive healthcare [4]. The relatively low- cost computation, high performance, robustness, generalization ability, and high accuracy have been often associated with machine learning methods [5].

Common methods such as angiography [6] for diagnosing is costly and has adverse side effects. Consequently, the healthcare industry has been investing on computer-aided disease diagnosis methods such as machine learning. Whereas, the data mining process by utilizing machine learning science and database management knowledge [1] has become a robust tool for data analysis and management of big data which ultimately leads to knowledge extraction. It should be noted that with the progress of health informatics, including industry 4.0, the healthcare systems have been more intelligent, automated, and equipped with early diagnosis, warning systems, and predictive strategies [7]. In this new generation, with the development of new medical devices, equipment, and tools, new knowledge can be gained in the field of disease diagnosis. One of the best ways to quickly diagnose diseases is to use computer-assisted decision making, i.e., machine learning to extract knowledge from data. In general, knowledge extraction from data is an approach that can be very crucial for the medical industry in diagnosing and predicting diseases. In other words, the purpose of knowledge extraction is the discovery of knowledge from databases in the data mining process. Data mining is used as a suitable approach to reduce costs and for quick diagnosis of the disease.

**Figure 1:**
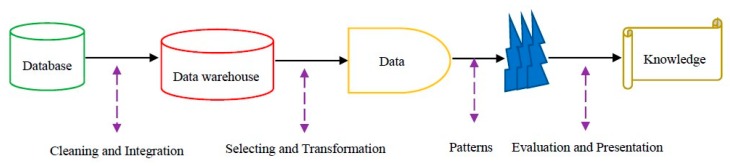
Database knowledge discovery (KDD) process steps [1].

Therefore, the purpose of the data mining process, known as database knowledge discovery (KDD), is to find a suitable pattern or model of data that was previously unknown so that these models can be used for specific disease diagnosis decisions in the healthcare environment [1]. Steps of the KDD process [1] include data cleaning (to remove disturbed data and conflicting data) and data integration (which may combine multiple data sources), data selection (where appropriate data is retrieved from the database for analysis), and data transformation (where data are synchronized by performing summary or aggregation operations and transformed appropriately for exploring), data mining (the essential process in which intelligent methods are used to extract data patterns), pattern evaluation (to identify suitable patterns that represent knowledge based on fit measurements), and knowledge presentation (where visualization and presentation techniques are used to provide users with explored knowledge) are shown in Figure 1.

The subject of this study is in the field of heart disease. Heart disease encompasses a variety of conditions, including congenital diseases, coronary artery disease, and heart rheumatism. Among these conditions, coronary artery disease is the most common, therefore, comprehensive reports of heart disease have been conducted in recent years on heart disease. The World Health Organization (WHO) has declared coronary artery disease (CAD) as the most common type of cardiovascular disease [8]. More than 30% of deaths worldwide were due to CAD, which resulted in more than 17 million deaths in 2015 [9]. Additionally, more than 360,000 Americans have died from heart attacks [10]. As a result, heart disease costs alone total more than $200 billion in the United States annually [10]. In addition, health care costs for heart disease will double by 2030, according to the American Heart Association [11].

Hence, in this paper, attention has been paid to a heart disease case study in order to apply a prediction method to coronary artery disease [6,12]. One way to accurately diagnose this disease is to use data mining methods to build an appropriate and robust model that is more reliable than medical imaging tools, including angiography in the field of diagnosis of coronary heart disease [4,5,6]. A main challenge in model learning is the feature selection problem, as the feature selection step is so important in data mining and its purpose is to eliminate unnecessary and unimportant features [1,13,14,15]. The method used in this study is the feature ranking-selection method to choose the best subset of features in dataset. In this method, we utilize various data mining methods including random trees (RTs), decision tree of C5.0, Chi-squared automatic interaction detection (CHAID), and support vector machine (SVM). Through these methods, the selection of the subset of features according to their order of priority takes place. For this purpose, the subset of features is ranked from the least important to the most important due to the different weightings to the features associated with the classification models that these features were assigned to in the output simulator.

Finally, among the classification models used in this study, obtaining the most appropriate subset feature by random trees model with the best classification set and the most accurate classification of coronary-heart disease diagnosis is the main purpose of this study. As a result, in terms of accuracy, area under the curve (AUC) and Gini value criteria for CAD diagnosis, random trees model is the best model compared to other prediction models.

The rest of the paper is organized as follows: data mining classification methods are presented in Section 2 and related works are described in Section 3. The proposed methodology is explained in Section 4. Section 5 represents the evaluated results of the experiment. Section 6 presents findings of the research and the conclusions, namely “Results and Discussion” and “Conclusions” in Section 7.

## 2. Data Mining Classification Methods

In this section, we describe the classification methods used in this study. These methods include CHAID decision tree, C5.0 decision tree, random trees, and support vector machine (SVM). Among the mentioned methods, except for the support vector machine, CHAID, C5.0, because random trees (RTs) are based on the decision tree, rules are extracted that are useful for the diagnosis of CAD, especially rule extraction using RTs.

### 2.1. Decision Tree of CHAID

The Chi-squared automatic interaction detection (CHAID) is one of the oldest tree classifications, and it is a supervised learning method by building the decision tree, which is evidence of the rules extraction, which is proposed by Kass [16]. This classification model is a statistical method based on the diagnosis of Chi-squared automatic interaction, and it is a recessive partitioning method that can be obtained by input features as predictors and the predictive class, a Chi-squared statistic test between target class and the predictive features are computed [17,18,19] so that the predictive features are ranked in order of their priority. As such, the most significant predictors of subset feature with the highest probability of their weight to diagnose CAD can be gained. It should be noted that the process of selecting a significant predictor feature is based on data sample segmentation so that until we reach an external node i.e., the leaf, the samples partition continues into smaller subdivisions [17,20].

In general, the CHAID model includes the following steps [17,18,19]:

1. Reading predictors: the first step is to make classified predictors or features out of any consecutive predictors by partitioning the concerned consecutive disseminations into a number of classifiers with almost equal numbers of observations. For classified predictors, the classifiers or target classes are determined.
2. Consolidating classifiers: the second step is to round through the features to estimate for each feature the pair of feature classifiers that is least significantly different with regard to the dependent variable. In this process, the CHIAD model includes two types of statistical tests. One, for the classification dataset, it will gain a Chi-square test or Pearson Chi-square. The assumptions for Chi-square test are as follows:

*N_ij_* = The value of observations concerned with feature fields or sample size,

*G_ij_* = The gained expected feature fields for datasets, for example, the training dataset (xn = i, yn = j),

*V_n_* = The value weight (*W_n_*) concerned with per sample of dataset,

*D_f_* = The most number of logically independent values, which are values that have the freedom to vary, in the dataset, namely, Degrees of Freedom. *D_f_* is equal to (*Nij*−*1*).

*C* = The corresponsive data sample, afterward:

(1) $$X^{2}=\sum_{j=1}^{j}\sum_{i=1}^{D}\frac{{\left(N_{ij}-G_{ij}\right)}^{2}}{G_{ij}},$$

(2) $$N_{ij}=\sum_{N\epsilon C}F_{n}D_{f}\left(X_{n}=i\;\cap \;Y_{n}=j\right).$$

Two, for regression datasets where the dependent variable is consecutive, for measure-dependent variables, *F*-tests are used. If the concerned test for a given pair of feature classifiers is not statistically significant as defined by an alpha-to-consolidate value, then it will consolidate the concerned feature classifiers and iterate this step, i.e., obtain the next pair of classifiers, which now may include previously consolidated classifiers. If the statistical significance for the concerned pair of feature classifiers is significant, i.e., less than the concerned alpha-to-consolidate value), then it will gain optionally a Bonferroni adopted *p*-value for the set of classifiers for the concerned feature.

(3) $$F=\frac{\sum_{i=1}^{D}\sum_{N\epsilon C}W_{n}V_{n}D_{f}\left(X_{n}=i\right){\left(Y_{i}^{\prime }-Y\right)}^{2}/(D_{f}-1)}{\sum_{i=1}^{D}\sum_{N\epsilon C}W_{n}V_{n}D_{f}\left(X_{n}=i\right){\left(Y_{n}-Y^{\prime }\right)}^{2}/(N_{f}-D_{f})},$$

given that the functions *Y_n_*, *Y*, and *N_f_* are formulated as follows:

(4) $$Y_{n}=\frac{\sum_{N\epsilon C}W_{n}V_{n}Y_{n}D_{f}\left(X_{n}=i\right)}{\sum_{N\epsilon C}W_{n}V_{n}D_{f}\left(X_{n}=i\right)},$$

(5) $$Y=\frac{\sum_{N\epsilon C}W_{n}V_{n}Y_{n}D_{f}}{\sum_{N\epsilon C}W_{n}V_{n}D_{f}},$$

(6) $$N_{f}=\underset{N\epsilon C}{\sum }V_{n}.$$

3. Selecting the partition variable: the third step is to select the partition of the predictor variable with the smallest adapted *p*-value, i.e., the predictor variable that will gain the most significant partition. The *p*-value is formulated in a $\left(P=pr\left(X_{c}^{e}>X^{2}\right)\right)$. If the smallest (Bonferroni) adopted *p*-value for any predictor feature is greater than some alpha-to-partition value, then no further partitions will be done, and the concerned node is a final node. This process is continued until no further partitions can be done, i.e., given the alpha-to-consolidate and alpha-to-partition values). Eventually, according to step 2, the *p*-value is obtained as follows:
  (7) $$P=P\left(F\left(C-1,\;N_{f}-1\right)>F\right).$$

### 2.2. Decision Tree of C5.0

Following is the process of improving decision tree models including ID3 [21,22], C4.5 [23,24,25], the C5.0 tree model [26,27,28,29] as the latest version of decision tree models developed by Ross. The improved C5.0 decision tree is manifold faster than its ally models in terms of speed. In terms of memory usage, the memory gain in this model is much higher than the other models mentioned. The model also improves trees by supporting boosting and bagging [25] so that using it increases accuracy of diagnosis. As one of the common characteristics among decision trees is weighting of disease features, but the C5.0 model allows different features and types of incorrect classifies to be weighted.

One of the crucial advantages of the C5.0 model to test the features is the gain ratio, with increased information gain, i.e., the information entropy, and the bias is reduced [1,17,29]. For example, the assumptions for the information entropy, information gain, and gain ratio problems are as follows [1,17,25]:

We assume the *S* as a set of training dataset and split *S* into *n* subsets, and, *N_i_* = the sample dataset of *K* features.

Therefore, we obtain the features to diagnosis CAD selected with the least information entropy, and the most information gain and gain ratio. The information entropy, information gain, and gain ratio are formulated as follows.

(8) $$Info_{\;Gain}\left(S,K\right)=\overset{N\in C_{i}}{\underset{i=1}{\sum }}P_{i\;}\times Info_{\;Entropy}\left(S_{i}\right),$$

(9) $$Info{\;}_{Entropy}\left(S\right)=\;-\;\overset{N\in C_{i}}{\underset{j=1}{\sum }}P_{i}{log}_{2}P_{i}.$$

Based on Equations (8) and (9), the number of *K* features, a partition *S* according to values of *K*, and where *P* is the probability distribution of division (*C1*, *C2*, …, *Ci*):

(10) P=(|C1|/|S|, |C2|/|S|, …, |Ci|/|S|).

Based on Equation (10), *Ci* is the number of disjoint classes and |S| is the number of samples in set of *S*. The value of Gain is computed as follows.

(11) $$Gain\;\left(S,K\right)=\;Info{\;}_{Entropy}\left(S\right)-Info_{\;Gain}\left(S,K\right).$$

Ratio instead of gain was suggested by Quinlan so that *Split Info (K,C)* is the information due to the division of *C* on the basis of value of categorical feature *K*, using the following:

(12) $$Split\;Info\left(K,C\right)=\;Info{\;}_{entropy}\left(\frac{\left|C1\right|}{\left|C\right|},\frac{\left|C2\right|}{\left|C\right|},\;\ldots ,\frac{\left|Ci\right|}{\left|C\right|}\right),$$

(13) GainRatio(K,C) = Gain(K,C) / Split Info(K,C).

For Equations (12) and (13), where (C1, C2, … Ci) is the partition of C induced by value of K.

### 2.3. Support Vector Machine

**Figure 2:**
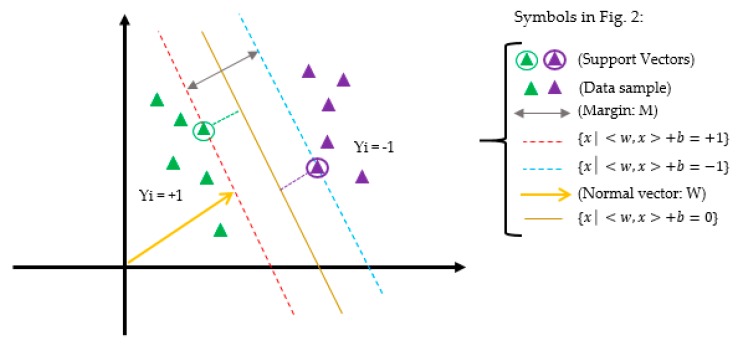
Support vector machine in two-dimensional space.

Support Vector Machine (SVM) is a supervised learning model based on statistical learning theory and structural risk minimization [29,30] presented by Vapnic that only the data assigned in the support vectors are based on machine learning and model building. The SVM model is not sensitive to other data points and its aim is to find the best separation line, i.e., the optimal hyperplane between the two classes of samples so that it has the maximum distance possible to all the two classes of support vectors [29,30,31,32]. The predictor feature is determined by the separator line for each predictive class. Figure 2 shows the scheme of the support vector machine in two-dimensional space.

Regarding Figure 2, a description of SVM model is as follows: 

Allow training data sample $\left\{\left(xi,\;yi\right)\right\}i=1\ldots n,\;xi\in R^{d}$ and the data of the two classes labeled yi ∈ {−1, 1} to be separated by a optimal hyperplane in a {x│〈w,x〉+b=0} so it is assigned in the middle of the other two lines, i.e., {x│〈w,x〉 + b = +1} and {x│〈w,x〉 + b=−1} with margin *M* that the margin $\left(M=\frac{2}{∥W∥}\right)$ of the separator is the distance between support vectors, data samples closest to the hyperplane are support vectors, and also, *b* represents the offset between the optimal hyperplane and the origin plane. Then for each training sample (xi, yi):

(14) $$\left\{\begin{array}{c} W^{T}X_{i}\;+\;b\;\leq \;-\frac{M}{2}\;if\;yi\;=\;-1\\ W^{T}X_{i}\;+\;b\;\geq \;\frac{M}{2}\;if\;yi\;=\;1 \end{array}\right\}↔Y_{i}\left(W^{T}X_{i}+b\right)\geq \frac{M}{2}.$$

According to the hyperplane optimization that the SVM model was meant to solve, the optimization problem is as follows [29]:

(15) $$Minimize:\;\frac{1}{2}\;∥W{∥}^{2}\;subjected\;to:\;y_{i}\left(w.x+b\right)-1\geq 0\;\forall i.$$

To solve the problem of Equation (15), one has to obtain the dual of the problem using the Lagrange Method, namely, (*Lp*). To obtain the dual form of the problem, the nonnegative Lagrangian coefficients are multiplied by αi ≥ 0. L_p_ is defined as follows:

(16) $$L_{p}=\frac{1}{2}\;{\left|w\right|}^{2}-\sum_{i}\;\alpha_{i}\;(y_{i}\left(w.x+b\right)-1.$$

Finally, Equation (16) is transformed into the following equation [29]:

(17) $$\mathrm{Maximize}\;:L_{D}=\sum_{i}\alpha_{i}-\frac{1}{2}\sum_{i}\sum_{j}\alpha_{i}\alpha_{j}Y_{i}Y_{j}\left(X_{i}X_{j}\right).$$

Equation (17) is called the dual problem, namely, *L_D_*. However, for nonlinear SVM due to the absence of trade-off between maximizing the margin and the misclassification. Therefore, it could not obtain the linear separate hyperplane in the data sample. In the nonlinear space, the best solution, the basic data of higher dimension, i.e., feature space, of the linear separate is transformed. At the end, kernel functions are used, such as linear, polynomial, radial basis function (RBF), and sigmoid [29]. Based on Equation (18), L_D_ for the nonlinear data sample is obtained. In Equation (18), parameter *C* is the penalty agent and determines the measure of penalty placed to a fault, so that the “*C*” value is randomly selected by the user.

(18) $$\begin{array}{c} \mathrm{Maximize}\;\Phi \left(W,b,\xi ,\alpha ,\beta \right):L_{D}=\overset{N}{\underset{i=1}{\sum }}\alpha_{i}-\frac{1}{2}\overset{N}{\underset{i=1}{\sum }}\overset{N}{\underset{j=1}{\sum }}\alpha_{i}\alpha_{j}Y_{i}Y_{j}K\left(X_{i},X_{j}\right)\\ \mathrm{subjected}\;\mathrm{to}\;\sum_{j}\alpha_{j}Y_{j}=0,\;0\leq \alpha_{i}\leq C,\;i=1,\ldots ,N. \end{array}$$

*N* is the number of data samples in Equation (18). In this study, the radial basis function (RBF) [29] is selected as the kernel function as shown in Equation (19):

(19) $$K\left(X_{i},X_{j}\right)=\;\exp(-ϒ∥X_{i}-X_{j}{∥}^{2}.$$

In Equation (19), the kernel parameter of ϒ with respect to (ϒ≥0) represents the width of the RBF.

### 2.4. Random Trees

The model of random trees (RTs) is one of the robust predictive models, better than other classification models in terms of accuracy computing, data management, more information gain with eliminating fewer features, extracting better rules, working with more data, and more complex networks. Therefore, the model for disease diagnosis is suitable. This model consists of multiple trees randomly with high depth so that the most significant votes are chosen from a set of possible trees having *K* random features at each node. In other words, in the set of trees, each tree has an equal probability of being assigned. Due to the experiments performed in the classification of the dataset, the accuracy of the RTs model is more accurate than the other models because it uses the evaluation of several features and composes functions. Therefore, RTs can be constructed efficiently and the combination of large datasets of random trees generally leads to proper models. There has been vast research in recent years of RTs in the field of machine learning [33]. Generally, random trees is confirmed as a crucial performance as compared to the classifiers presented as a single tree in this study.

If we consider random trees at very high dimensions with a complex network, then it can include the following steps [33,34]:

1. Using the *N* data sample randomly, in the training dataset to develop the tree.
2. Each node as a predictive feature grasps a random data sample selected so that *m* < *M* (*m* represents the selected feature and *M* represents the full of features in the corresponding dataset. Given that during the growth of trees, *m* is kept constant.)
3. Using the *m* features selected for generating the partition in the previous step, the *P* node is computed using the best partition path from points. *P* represents the next node.
4. For aggregating, the prediction dataset uses the tree classification voting from the trained trees with *n* trees.
5. For generating the terminal RTs, the model uses the biggest voted features.
6. The RTs process continues until the tree is complete and reaches only one leaf node.

## 3. Related Works

In recent years, several studies have been conducted on the diagnosis of CAD on different datasets using data mining methods. The most up-to-date dataset that researchers have used recently is the Z-Alizadeh Sani dataset in the field of heart disease. To this end, we review recent research on the Z-Alizadeh Sani dataset [35,36].

Alizadeh Sani et al. have proposed the use of data mining methods based on ECG symptoms and characteristics in relation to the diagnosis of CAD [37]. In their research, they used sequential minimal optimization (SMO) and naïve Bayes algorithms separately and in combination to diagnose the disease. Finally, using the 10-fold cross-validation method for the SMO-naïve Bayes hybrid algorithm, they achieved greater accuracy of 88.52% than the SMO (86.95%) and naïve Bayes (87.22%) algorithms.

In another study, Alizadeh Sani et al. developed classification algorithms such as SMO, naïve Bayes, bagging with SMO, and neural networks for the diagnosis of CAD [12]. Confidence and information gain on CAD have also been used to determine effective features. As a result, among these algorithms, SMO algorithm with information gain has the best performance, with accuracy of 94.08% using the 10-fold cross-validation method.

Alizadeh Sani et al. have used computational intelligence methods to diagnose CAD, and they have separately diagnosed three major coronary stenosis using demographics, symptoms and examination, ECG characteristics, laboratory analysis, and echo [38]. They have used analytical methods to investigate the importance of vascular stenosis characteristics. Finally, using the SVM classification model with 10-fold cross-validation method, along with feature selection of combined information gain and average information gain, they obtained accuracies of 86.14%, 83.17%, and 83.50% for left anterior descending (LAD), left circumflex (LCX), and right coronary arteries coronaries (RCA), respectively.

Arabasdi et al. have presented a neural network-genetic hybrid algorithm for the diagnosis of CAD [39]. For this purpose, in their research, genetic and neural network algorithms have been used separately and a hybrid to analyze the dataset, and the accuracy of the neural network algorithm and neural network-genetic algorithm using the 10-fold cross-validation method was 84.62% and 93.85%, respectively.

Alizadeh Sani et al. have performed a feature engineering algorithm that used the naïve Bayes, C4.5, and SVM classifiers for non-invasive diagnosis of CAD [36]. They increased their dataset from 303 records to 500 samples. The accuracy obtained using the 10-fold cross-validation method for naïve Bayes, C4.5, and SVM algorithms were 86%, 89.8%, and 96.40%, respectively.

In a study conducted by Abdar et al. [40], the authors used a two-level hybrid genetic algorithm and NuSVM called N2Genetic-NuSVM. The two-level genetic algorithm was used to optimize the SVM parameters and to select the features in parallel. Using their proposed method, the accuracy of CAD diagnosis was 93.08% through a 10-fold cross-validation method.

## 4. Proposed Methodology

**Figure 3:**
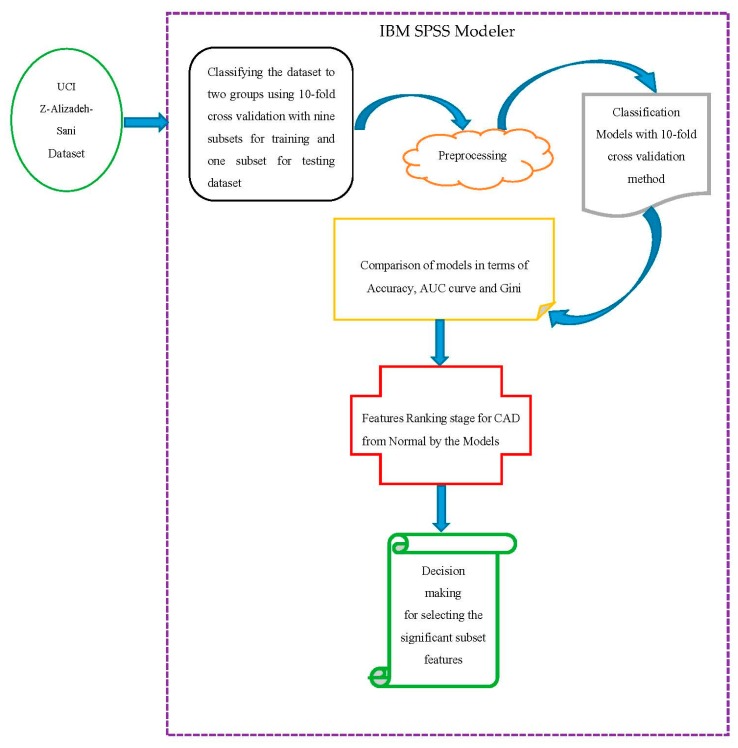
Proposed methodology.

In this section, we follow the proposed methodology in Figure 3 by using IBM Spss Modeler version 18.0 software for implementation of classification models.

### 4.1. Description of the Dataset

Initially based on Figure 3, to diagnose the CAD, the Z-Alizadeh Sani dataset was used in this study [35]. This dataset contains information on 303 patients with 55 features, 216 patients with CAD, and 88 patients with normal status. The features used in this dataset were divided into four groups that were features of CAD for patients including demographics, symptoms, and examination, electrocardiogram (ECG), and laboratory and echo features, described in Table 1. For categorizing the CAD from Normal, the diameter narrowing above 50% represents a patient as CAD, and its absence is stated as Normal [12].

### 4.2. Classifying the Dataset

Data was classified into nine subsets i.e., 90% for training the classifiers and one subset i.e., 10% for testing dataset using 10-fold cross-validation.

### 4.3. Preprocessing the Dataset

The preprocessing step was performed after the data was classified. In general, a set of operations lead to the creation of a set of cleaned data that can be used on the dataset, investigation operation, so-called data preprocessing. The samples values in the Z-Alizadeh-Sani dataset [35] were numeric and string. The purpose of preprocessing the data in this study was to homogenize them so that all data was in the domain of (0, 1), which is called the normalization operation, so that the standard normalization operation was employed using the Min-Max function. After normalizing numbers, the string data was transformed to numeric. In this regard, given the nature of the string data, the value was assigned to them in the interval (0, 1). For example, the sex feature had male and female values that transformed to zero and one, respectively.

### 4.4. Classifying the Models Using the 10-Fold Cross-Validation Method

For classifying the models the 10-fold cross-validation method was used [41], where the dataset was randomly divided into the same K-scale for the division so and the k-1 subset being used to train the classifiers. The rest of the division was also used to investigate the output performance at each step, and repeated 10 times. For this purpose, classification of the prediction models was performed based on the 10-fold cross-validation method so that the average of the criteria was obtained 10-fold [1,42], as 90% of the data was used for training and 10% was used for testing the data. Finally, this cross-validation process was executed 10 times so that the results were demonstrated by averaging each 10 times.

## 5. Evaluating the Results

In this section, we examine the evaluation in two subdivisions. First, evaluation based on the classification criteria, including ROC curve, Gini, gain, confidence, return on investment (ROI), profit, and response. Second, the evaluation was based on significant predictive features.

### 5.1. Evaluation Based on Classification Criteria

We used a confusion matrix [1,39,43,44] to evaluate classification models such as SVM, CHAID, C5.0, and RTs in the diagnosis of CAD on the Z-Alizadeh Sani dataset described in Table 2.

**Figure 4:**
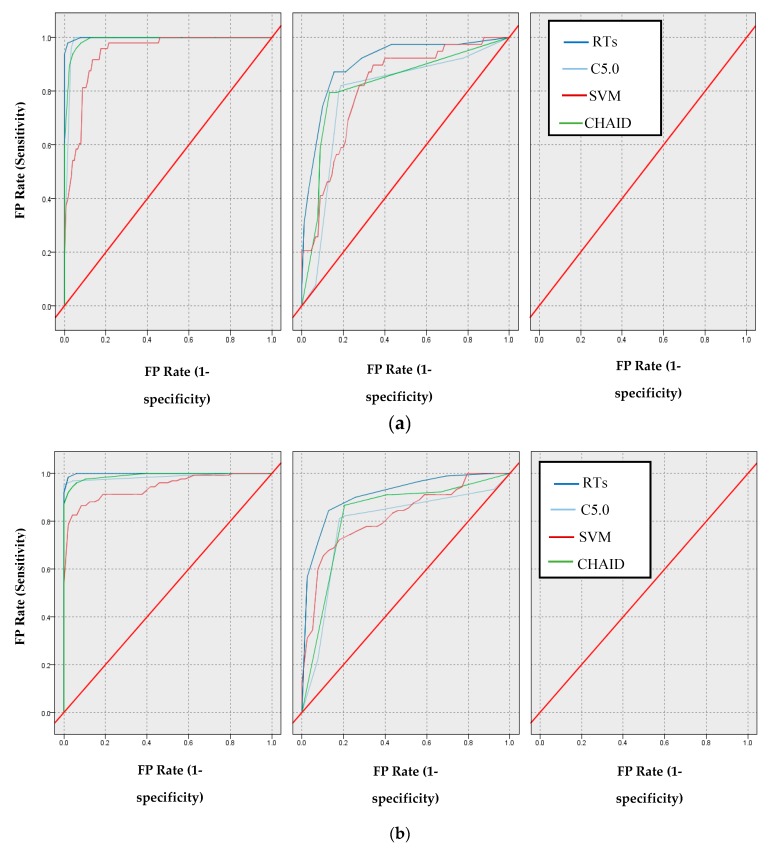
Comparison based on ROC of models: (**a**) Normal class (**b**) CAD class.

In the following, through the confusion matrix method, the AUC [1,45] and the Gini index [46] criteria were obtained, and the comparison between the models mentioned for this AUC criterion are shown in Figure 4a,b.

According to Figure 4b, the AUC values for the SVM, CHAID, C5.0, and RTs models are 80.90%, 82.30%, 83.00%, and 90.50%, respectively. Additionally, the Gini values for SVM, CHAID, and RTs models are 61.80%, 64.60%, 66.00%, and 93.40%, respectively.

**Figure 5:**
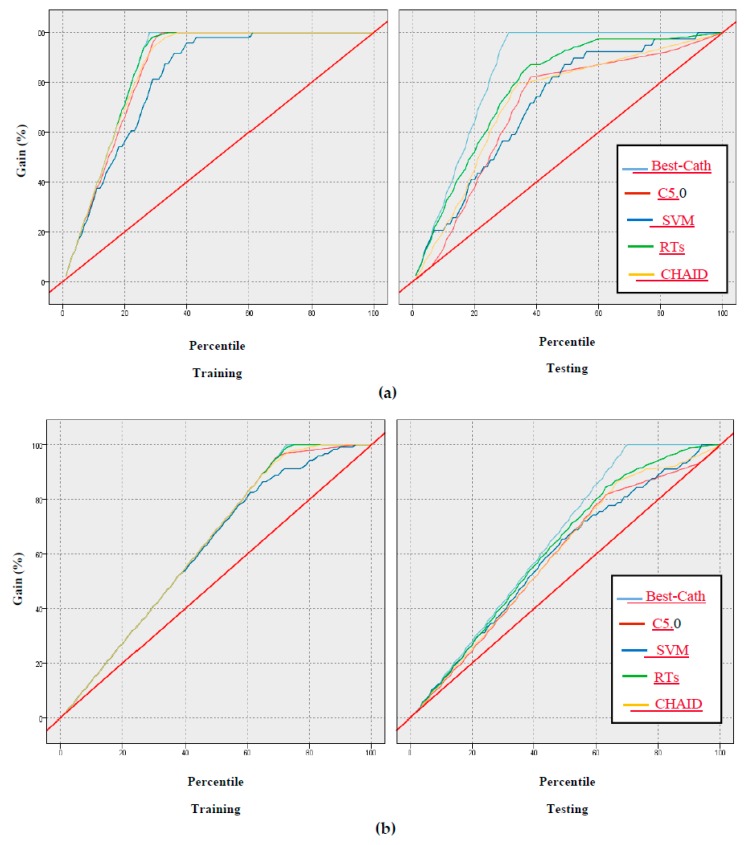
Results based on gain of models: (**a**) Normal class, (**b**) CAD class.

**Figure 6:**
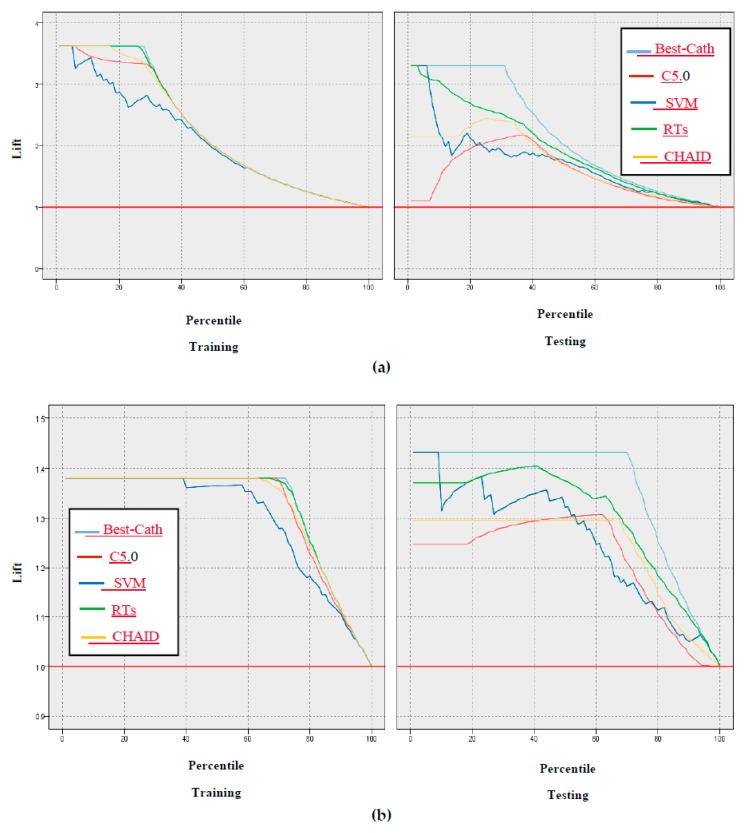
Results based on confidence through the lift chart of models: (**a**) CAD class, (**b**) Normal class.

**Figure 7:**
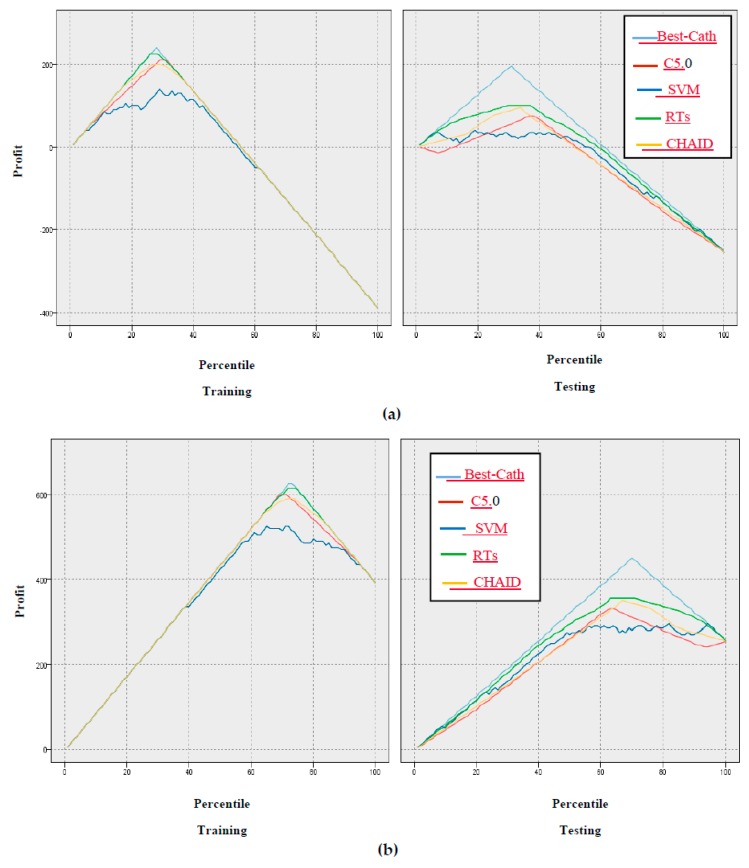
Results based on profit of models: (**a**) Normal class, (**b**) CAD Class.

**Figure 8:**
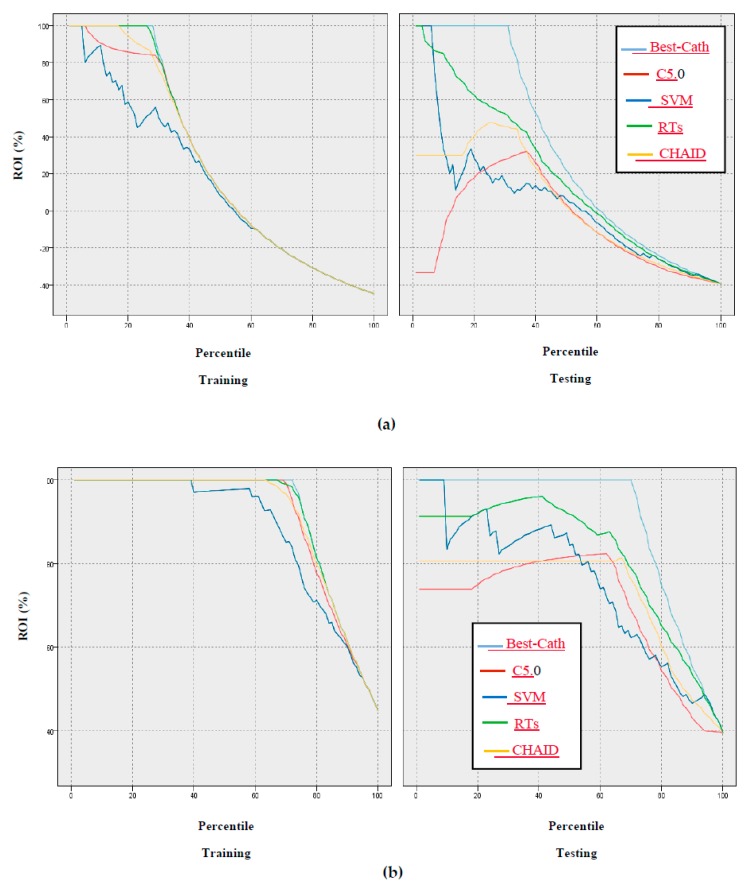
Results based on ROI of models: (**a**) CAD class, (**b**) Normal class.

**Figure 9:**
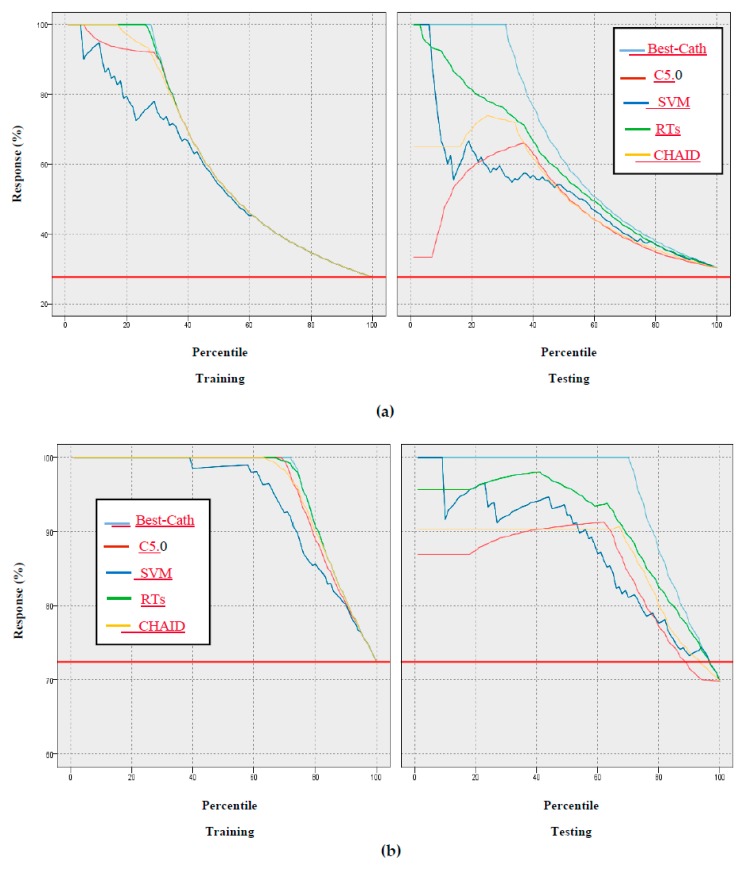
Results based on response of models: (**a**) CAD class, (**b**) Normal class.

In addition, the gain, confidence, profit, ROI, and response criteria for evaluating the models were examined, and comparisons between models through these criteria are shown in Figure 5, Figure 6, Figure 7, Figure 8 and Figure 9.

According to Figure 5, Figure 6, Figure 7, Figure 8 and Figure 9 of the criteria in the relevant models for the CAD diagnosis of the Normal class, it can be said that the RTs model has better performance in terms of gain, confidence, ROI, profit, and response criteria than other classification models.

### 5.2. Evaluation Based on Significant Predictive Features

One of the significant evaluations for comparing classification models for predicting the CAD from Normal is the use of the importance of predictive features. To this end, we examined the models in terms of their importance in the ranking stage of features. In fact, the models were measured according to the weight determined by the predictor features. The weighted importance of the features for the models is shown in Table 3, Table 4 and Table 5.

## 6. Results and Discussion

In the modeling process proposed in Section 4, we implemented several data mining models including SVM, CHAID, C5.0, and RTs. The 10-fold cross-validation method was used to build these models so that the data was divided into training (90%) and test (10%) subsets. The results show that the random trees model is the best classification model compared to the other models so that 91.47% accuracy of the RTs model was obtained using the 10-fold cross-validation method. While the accuracy of SVM, CHAID, and C5.0 models were 69.77%, 80.62%, and 82.17%, respectively.

The accuracy was computed using the following formula (TP + TN/(TP + TN + FP + FN)) where TP is true positive, TN is true negative, FP is false positive, and FN is false negative [1].

Another criterion for evaluating the models in this study was the AUC criterion, where 80.90%, 82.30%, 83.00%, and 96.70% was obtained for SVM, CHAID, C5.0, and RTs, respectively.

Furthermore, an achievement of this study was the use of criteria that were not found in previous studies, including gain, confidence, ROI, profit, and response, as shown in Figure 5, Figure 6, Figure 7, Figure 8 and Figure 9. In terms of this criteria, the random trees model has the best performance compared to the other classification models.

Finally, based on Table 3, Table 4, Table 5 and Table 6, it can be found that in each of the four models, the typical chest pain feature was selected as the most significant predictor so that the predictor significance of the typical chest pain feature for the random trees model was equal to 0.98, and the least significant for the lymph feature was equal to zero. Considering this, intervals 1 and 2 were applied in the simulator. In Table 1, typical chest pain was the most significant feature with a value of 0.04 and the region RWMA was the least significant feature with a value of 0.01. According to Table 4 and Table 5, typical chest pain was the most significant feature, equal to 0.28 and 0.33, respectively, and the least significant feature according to Table 4 for the EF-TTE feature was equal to zero and the least significant feature according to Table 6 was 0.02. It is therefore confirmed that the RTs model is the best model relative to other classification models according to the above tables.

One of the advantages of the random trees model is the most significant obtained rules of CAD diagnosis that are mentioned in Table 7.

According to Table 7, the extracted rules for CAD are described as follows:

If the condition is true of (BP > 110.0), (FH > 0.0), (Neut > 51.0), and (Typical Chest Pain > 0.0), then the CAD exist highly accurate and also interestingness index, otherwise, the person is Normal. If the condition is true of (Typical Chest Pain > 0.0) and (Atypical = {N}), then the result it is like the result of previous conditions. In the following, if the condition is true of (Weight > 8.0), (CR > 0.9), (Typical Chest Pain > 0.0), and (Atypical = {N}), then the person is abnormal or CAD. Finally, if (K ≤ 4.9), (WBC > 5700.0), (CR < 0.9), (DM > 0.0), and (Typical Chest Pain > 0.0) is true, as a result the person is abnormal.

In recent years, several studies have been conducted on the diagnosis of CAD on different datasets using data mining methods. To this end, we review recent research on the updated Z-Alizadeh Sani dataset, as described in Table 8. The results of accuracy, AUC, and Gini criteria for the models were obtained according to the 10-fold cross validation method compared to previous studies.

Taking a look at Table 8, it can be seen that the proposed method based on random trees outperforms other methods in terms of accuracy, AUC, and Gini criteria. It implies that the 40 features extracted by using RTs are the most informative ones about the CAD disease.

## 7. Conclusions

In this study, a computer-aided diagnosis system was used to diagnose CAD as a common heart disease on the Z-Alizadeh Sani dataset [35], and this system was implemented using the IBM Spss Modeler version 18.0 tool. Since angiography is the most common tool of diagnosis of heart disease, it has cost and side effects for individuals. Therefore, artificial intelligence methods, that is, machine learning techniques, can be a solution to the stated challenge. Hence, such classification models including SVM, CHAID, C5.0, and random trees were used for modeling with the 10-fold cross-validation method, that are based on accuracy, AUC, Gini, ROI, profit, confidence, response, and gain, and were examined and evaluated. Finally, based on the criteria stated, the random trees model was found as the best model compared to the other models. The predictive features were selected based on the order of their priority with the highest accuracy, and we concluded that the random trees model with the most significant features of 40 and the accuracy of 91.47% has better performance than the other classification models. Considering this, with this number of features, we had more information gain than the features selected in previous works. Another achievement of this study was the important extraction rules for CAD diagnosis using the random trees model, with these rules shown in Table 6. As future work, the fuzzy intelligent system can be used in combination with artificial intelligence models to diagnose CAD on the Z-Alizadeh Sani dataset and other datasets. Another way to better diagnose CAD disease on this dataset and other real datasets is deep learning models and combining deep learning approaches with distributed design and architecture.

## Figures and Tables

**Table 1:** Description of the features used in the Z-Alizadeh-Sani dataset with their valid ranges.

Feature Type	Feature Name	Range	Measurement			
			Mean	Std. Error of Mean	Std. Deviation	Variance
Demographic	Age	(30–80)	58.90	0.6	10.39	108
Demographic	Weight	(48–120)	73.83	0.69	11.99	143.7
Demographic	Length	(140–188)	164.72	0.54	9.33	87.01
Demographic	Sex	Male, Female	---	---	---	---
Demographic	BMI (body mass index Kb/m^2^)	(18–41)	27.25	0.24	4.1	16.8
Demographic	DM (diabetes mellitus)	(0, 1)	0.3	0.03	0.46	0.21
Demographic	HTN (hypertension)	(0, 1)	0.6	0.03	0.49	0.24
Demographic	Current smoker	(0, 1)	0.21	0.02	0.41	0.17
Demographic	Ex-smoker	(0, 1)	0.03	0.01	0.18	0.03
Demographic	FH (family history)	(0, 1)	0.16	0.02	0.37	0.13
Demographic	Obesity	Yes if MBI > 25, No otherwise	---	---	---	---
Demographic	CRF (chronic renal failure)	Yes, No	---	---	---	---
Demographic	CVA (cerebrovascular accident)	Yes, No	---	---	---	---
Demographic	Airway disease	Yes, No	---	---	---	---
Demographic	Thyroid disease	Yes, No	---	---	---	---
Demographic	CHF (congestive heart failure)	Yes, No	---	---	---	---
Demographic	DPL (dyslipidemia)	Yes, No	---	---	---	---
Symptom and examination	BP (blood pressure mm Hg)	(90–190)	129.55	1.09	18.94	358.65
Symptom and examination	PR (pulse rate ppm)	(50–110)	75.14	0.51	8.91	79.42
Symptom and examination	Edema	(0, 1)	0.04	0.01	0.2	0.04
Symptom and examination	Weak peripheral pulse	Yes, No	---	---	---	---
Symptom and examination	Lung rates	Yes, No	---	---	---	---
Symptom and examination	Systolic murmur	Yes, No	---	---	---	---
Symptom and examination	Diastolic murmur	Yes, No	---	---	---	---
Symptom and examination	Typical chest pain	(0, 1)	0.54	0.03	0.5	0.25
Symptom and examination	Dyspnea	Yes, No	---	---	---	---
Symptom and examination	Function class	1, 2, 3, 4	0.66	0.06	1.03	1.07
Symptom and examination	Atypical	Yes, No	---	---	---	---
Symptom and examination	Nonanginal chest pain	Yes, No	---	---	---	---
Symptom and examination	Exertional chest pain	Yes, No	---	---	---	---
Symptom and examination	Low TH Ang (low-threshold angina)	Yes, No	---	---	---	---
ECG	Rhythm	Sin, AF	---	---	---	---
ECG	Q wave	(0, 1)	0.05	0.01	0.22	0.05
ECG	ST elevation	(0, 1)	0.05	0.01	0.21	0.04
ECG	ST depression	(0, 1)	0.23	0.02	0.42	0.18
ECG	T inversion	(0, 1)	0.3	0.03	0.46	0.21
ECG	LVH (left ventricular hypertrophy)	Yes, No	---	---	---	---
ECG	Poor R-wave progression	Yes, No	---	---	---	---
Laboratory and echo	FBS (fasting blood sugar mg/dL)	(62–400)	119.18	2.99	52.08	2712.29
Laboratory and echo	Cr (creatine mg/dL)	(0.5–2.2)	1.06	0.02	0.26	0.07
Laboratory and echo	TG (triglyceride mg/dL)	(37–1050)	150.34	5.63	97.96	9596.05
Laboratory and echo	LDL (low-density lipoprotein mg/dL)	(18–232)	104.64	2.03	35.4	1252.93
Laboratory and echo	HDL (high-density lipoprotein mg/dL)	(15–111)	40.23	0.61	10.56	111.49
Laboratory and echo	BUN (blood urea nitrogen mg/dL)	(6–52)	17.5	0.4	6.96	48.4
Laboratory and echo	ESR (erythrocyte sedimentation rate mm/h)	(1–90)	19.46	0.92	15.94	253.97
Laboratory and echo	HB (hemoglobin g/dL)	(8.9–17.6)	13.15	0.09	1.61	2.59
Laboratory and echo	K (potassium mEq/lit)	(3.0–6.6)	4.23	0.03	0.46	0.21
Laboratory and echo	Na (sodium mEq/lit)	(128–156)	141	0.22	3.81	14.5
Laboratory and echo	WBC (white blood cell cells/mL)	(3700–18.000)	7562.05	138.67	2413.74	5,826,137.52
Laboratory and echo	Lymph (lymphocyte %)	(7–60)	32.4	0.57	9.97	99.45
Laboratory and echo	Neut (neutrophil %)	(32–89)	60.15	0.59	10.18	103.68
Laboratory and echo	PLT (platelet 1000/mL)	(25–742)	221.49	3.49	60.8	3696.18
Laboratory and echo	EF (ejection fraction %)	(15–60)	47.23	0.51	8.93	79.7
Laboratory and echo	Region with RWMA	(0–4)	0.62	0.07	1.13	1.28
Laboratory and echo	VHD (valvular heart disease)	Normal, Mild, Moderate, Severe	---	---	---	---
Categorical	Target class: Cath	CAD, Normal	---	---	---	---

**Table 2:** Confusion matrix for detection of coronary artery disease (CAD).

The Actual Class	The Predicted Class	
	Disease (CAD)	Healthy (Normal)
Positive	True Positive	False Positive
Negative	False Negative	True Negative

**Table 3:** Predictor significance for features based on ranking for the random trees model.

No.	Feature	Predictor Significance
1	Typical chest pain	0.98
2	TG	0.66
3	BMI	0.63
4	Age	0.58
5	Weight	0.54
6	BP	0.51
7	K	0.48
8	FBS	0.43
9	Length	0.37
10	BUN	0.3
11	PR	0.29
12	HB	0.26
13	Function class	0.25
14	Neut	0.25
15	EF-TTE	0.25
16	WBC	0.24
17	DM	0.23
18	PLT	0.2
19	Atypical	0.19
20	FH	0.18
21	HDL	0.16
22	ESR	0.16
23	CR	0.14
24	LDL	0.14
25	T inversion	0.13
26	DLP	0.13
27	Region RWMA	0.12
28	HTN	0.11
29	Obesity	0.1
30	Systolic murmur	0.09
31	Sex	0.09
32	Dyspnea	0.08
33	Current smoker	0.06
34	BBB	0.05
35	LVH	0.03
36	Edema	0.02
37	Ex-smoker	0.02
38	VHD	0.01
39	St depression	0.01
40	Lymph	0.0

**Table 4:** Predictor significance for features based on ranking for the support vector machine (SVM) model.

No.	Feature	Predictor Significance
1	Typical chest pain	0.04
2	Atypical	0.03
3	Sex	0.02
4	Obesity	0.02
5	FH	0.02
6	Age	0.02
7	DM	0.02
8	Dyspnea	0.02
9	Systolic murmur	0.02
10	St depression	0.02
11	HTN	0.02
12	LDL	0.02
13	Current smoker	0.02
14	DLP	0.02
15	BP	0.02
16	LVH	0.02
17	Nonanginal	0.02
18	Tin version	0.02
19	Length	0.02
20	Function class	0.02
21	BBB	0.02
22	VHD	0.02
23	CHF	0.02
24	PR	0.02
25	WBC	0.02
26	BUN	0.02
27	FBS	0.02
28	ESR	0.02
29	CVA	0.02
30	Thyroid disease	0.02
31	Lymph	0.02
32	Weight	0.02
33	CR	0.02
34	Airway disease	0.02
35	TG	0.02
36	CRF	0.02
37	Diastolic murmur	0.02
38	Low TH ang	0.02
39	Exertional CP	0.02
40	Weak peripheral pulse	0.02
41	Neut	0.02
42	PLT	0.02
43	St elevation	0.02
44	EF-TTE	0.02
45	K	0.02
46	BMI	0.02
47	Ex-smoker	0.02
48	Lung rates	0.02
49	HDL	0.02
50	Na	0.01
51	Edema	0.01
52	Q wave	0.01
53	HB	0.01
54	Poor R progression	0.01
55	Region RWMA	0.01

**Table 5:** Predictor significance for features based on ranking for the C5.0 model.

No.	Feature	Predictor Significance
1	Typical chest pain	0.28
2	CR	0.14
3	ESR	0.13
4	T inversion	0.1
5	Edema	0.09
6	Region RWMA	0.08
7	Poor R progression	0.04
8	Sex	0.03
9	DM	0.03
10	BMI	0.02
11	WBC	0.02
12	DLP	0.02
13	Length	0.01
14	Dyspnea	0.0
15	EF-TTE	0.0

**Table 6:** Predictor significance imported for features based on ranking for the Chi-squared automatic interaction detection (CHAID) model.

No.	Feature	Predictor Significance
1	Typical chest pain	0.33
2	Age	0.15
3	T inversion	0.11
4	VHD	0.1
5	DM	0.09
6	HTN	0.04
7	Nonanginal	0.03
8	BP	0.02
9	Region RWMA	0.02
10	HDL	0.02

**Table 7:** The most significant obtained rules for CAD diagnosis using random trees (top decision rules for ‘cath’ class).

Decision Rule	Most Frequent Category	Rule Accuracy	Forest Accuracy	Interestingness Index
(BP > 110.0), (FH > 0.0), (Neut > 51.0) and (Typical Chest Pain > 0.0)	CAD	1.000	1.000	1.000
(BMI ≤ 29.02), (EF-TTE > 50.0), (CR ≤ 0.9), (Typical Chest Pain > 0.0) and (Atypical = {N})	CAD	1.000	1.000	1.000
(Weight > 8.0), (CR > 0.9), (Typical Chest Pain > 0.0) and (Atypical = {N})	CAD	1.000	1.000	1.000
(K ≤ 4.9), (WBC > 5700.0), (CR < 0.9),	CAD	1.000	1.000	1.000
(DM > 0.0) and (Typical Chest Pain > 0.0)	CAD	1.000	1.000	1.000

**Table 8:** The performed works for CAD diagnosis on the Z-Alizadeh Sani dataset with the 10-fold cross validation method.

Referense	Methods	No. Features Subset Selection	Accuracy (%)	Auc %	Gini %
[37]	Naïve Bayes-SMO	16	88.52	Not reported	Not reported
[12]	SMO along with information Gain	34	94.08	Not reported	Not reported
[38]	SVM along with average information gain and also information gain	24	86.14 for LAD 83.17 for LCX 83.50 for RCA	Not reported	Not reported
[39]	Neural network-genetic algorithm-weight by SVM	22	93.85	Not reported	Not reported
[36]	SVM along with feature engineering	32	96.40	92	Not reported
[40]	N2Genetic-nuSVM	29	93.08	Not reported	Not reported
In our study	Random trees	40	91.47	96.70	93.40
